# Effect of Counterclockwise Mandibular Autorotation on Temporomandibular Joint Spaces and Condylar Morphology After Bimaxillary Orthognathic Surgery: A CBCT-Based Study

**DOI:** 10.3390/jcm15031296

**Published:** 2026-02-06

**Authors:** Mehmet Emre Yurttutan, Merve Berika Kadıoğlu, Meyra Durmaz, Mehmet Alp Eriş, Mahzun Yıldız, Ömer Faruk Kocamaz

**Affiliations:** 1 Department of Oral and Maxillofacial Surgery, Faculty of Dentistry, Ankara University, 06500 Ankara, Türkiye; mehmet.alp.eris@gmail.com (M.A.E.); mzunyildiz22@gmail.com (M.Y.); omerf.kocamaz@gmail.com (Ö.F.K.); 2 Department of Orthodontics, Faculty of Dentistry, Ankara University, 06500 Ankara, Türkiye; mkadioglu@ankara.edu.tr (M.B.K.); dtmeyradurmaz@gmail.com (M.D.)

**Keywords:** dentofacial deformities, counterclockwise rotation, temporomandibular joint, orthognathic surgery, virtual surgical planning

## Abstract

**Background/Objectives:** Mandibular counterclockwise (CCW) autorotation following maxillary repositioning is a common biomechanical consequence of bimaxillary orthognathic surgery. However, its effect on temporomandibular joint (TMJ) morphology remains controversial. This study aimed to evaluate whether condyle-centered CCW mandibular autorotation influences postoperative TMJ spaces and condylar morphology using cone-beam computed tomography (CBCT). **Methods:** A total of 24 patients who underwent combined Le Fort I osteotomy and bilateral sagittal split ramus osteotomy were included in this retrospective analysis. Patients were divided into two groups based on virtual surgical planning: those with condyle-centered CCW autorotation (4–7°) and those without autorotation. Preoperative and one-year postoperative CBCT images were analyzed. Sagittal and coronal joint spaces, condylar dimensions, and glenoid fossa thickness were measured. Intra- and intergroup comparisons were performed using nonparametric statistical tests (α = 0.05). **Results:** Both groups demonstrated significant postoperative reductions in condylar height, width, and depth, reflecting adaptive bone remodeling. Joint space changes were limited overall. A significant intergroup difference was observed only in the change in the right superior joint space (*p* = 0.024), which decreased in the non-autorotation group but was preserved or slightly increased in the autorotation group. No other joint space or fossa parameter showed significant between-group differences. **Conclusions:** Condyle-centered CCW mandibular autorotation during bimaxillary orthognathic surgery does not induce adverse TMJ morphological changes beyond physiological adaptation. Preservation of the superior joint space suggests that autorotation may contribute to maintaining a more favorable condyle–fossa relationship. Incorporating controlled mandibular autorotation into surgical planning may support TMJ biomechanical balance and postoperative joint stability.

## 1. Introduction

Dentofacial deformities (DFDs) are significant disturbances that arise in the normal morphological proportions of the maxillomandibular complex and may affect facial aesthetics, occlusal relationships, and temporomandibular joint (TMJ) function [1]. In severe forms of DFDs, orthodontic treatment may be insufficient; therefore, treatment approaches involving orthognathic surgery, which aims to reposition the jawbones three-dimensionally, are implemented.

Orthognathic surgery involves the fixation of mandibular and/or maxillary bone segments into their preplanned new positions following osteotomy. Among the most common techniques in orthognathic surgery are bilateral sagittal split ramus osteotomy (BSSRO), developed by Obwegeser and Trauner [2], and Le Fort I osteotomy, which was derived from the maxillary fracture lines described by René Le Fort in 1901 and later rendered surgically applicable by Wassmund and Obwegeser [3,4,5].

Repositioning of the maxilla may lead to counterclockwise (CCW) autorotation of the mandible around its own axis to adapt to the new occlusal plane [6]. This autorotation may lead to significant biomechanical consequences in terms of the stability of the maxillomandibular relationship, occlusal harmony, and load distribution on the TMJ following surgery [7]. It has been reported that the increased joint load resulting from CCW rotation of the proximal segment, as well as the sagittal-plane rotation of the condyle, may be associated with condylar resorption and relapse. Furthermore, significant changes—particularly in the transverse axis—have been observed in condylar rotation, and these alterations are noted to potentially exert adverse effects on the TMJ [8,9]. However, studies have reported that if the condyle–disc relationship is anatomically normal, such load or rotational changes may not lead to resorption and that performing mandibular rotation centered on the condyle contributes to preserving TMJ adaptation by enhancing surgical stability [10,11]. In the literature, condylar displacement occurring within the physiological limits of the adaptive mechanism does not lead to morphological changes in the TMJ or the development of dysfunction [12].

Previous studies have demonstrated that radiological evaluation of the TMJ can be performed using various techniques, such as panoramic radiography, computed tomography (CT), and magnetic resonance imaging (MRI). Cone-beam computed tomography (CBCT) is considered a valuable imaging tool in the context of orthognathic surgery. Owing to its three-dimensional imaging capability, CBCT enhances diagnostic accuracy and allows for detailed assessment of bony structures and the spatial relationships of the condyles. With this method, the condylar position and angulations can be analyzed with high precision in sections obtained from different planes [13,14,15].

The aim of this study was to retrospectively evaluate changes in the condylar position and joint spaces in the TMJ via preoperative and postoperative CBCT images in individuals with dentofacial deformities who underwent BSSRO and Le Fort I surgery and to investigate whether mandibular autorotation leads to significant differences in TMJ morphology.

## 2. Materials and Methods

### 2.1. Study Design and Ethical Approval

Patient records from the Departments of Orthodontics and Oral and Maxillofacial Surgery at the Ankara University Faculty of Dentistry were retrospectively reviewed to identify patients who underwent bimaxillary orthognathic surgery (Le Fort I osteotomy and BSSRO) between 2020 and 2024. The study was approved by the Ankara University Clinical Research Ethics Committee (Decision No: 14/2, Date: 6 October 2025). Consent for the use of postoperative data was obtained from all participants through informed consent forms routinely collected prior to surgery.

Patients who underwent bimaxillary orthognathic surgery with virtual surgical planning (NemoFAB^®^ software, https://synapse-emea.fujifilm.com/; Nemotec, Madrid, Spain) at Ankara University Faculty of Dentistry; who had available preoperative and postoperative first-year CBCT records; who had no systemic or genetic diseases, no pathologies that could affect TMJ function, no history of trauma to the maxillofacial region, no cleft lip/palate, or other congenital deformities; and who had not previously undergone orthognathic surgery were included in the study.

Individuals with missing preoperative or postoperative CBCT records, those with a history of prior orthognathic surgery or orthodontic treatment, and those presenting with condylar deformities due to trauma or pathology were excluded from the study. Additionally, patients with advanced TMJ dysfunction or joint disease requiring surgical intervention, as well as those in whom artifacts or imaging distortions that could impede accurate measurements were present, were not included in the evaluation.

The authors employed ChatGPT 5.2 and Rubriq during manuscript preparation for translation assistance and language refinement. All AI-assisted content was thoroughly evaluated and revised by the authors, who retain full responsibility for the integrity and accuracy of the final work.

### 2.2. Power Analysis and Sample Selection

To determine the sample size of the study, a prospective power analysis was performed via G*Power (3.1.9.7) software. In the analysis, the effect size (f) was set at 0.305, the significance level (α) was 0.05, and the target power (1 − β) was 0.80. Accordingly, including 12 participants in each group (a total of 24 individuals) would provide sufficient statistical power.

**Figure 1:**
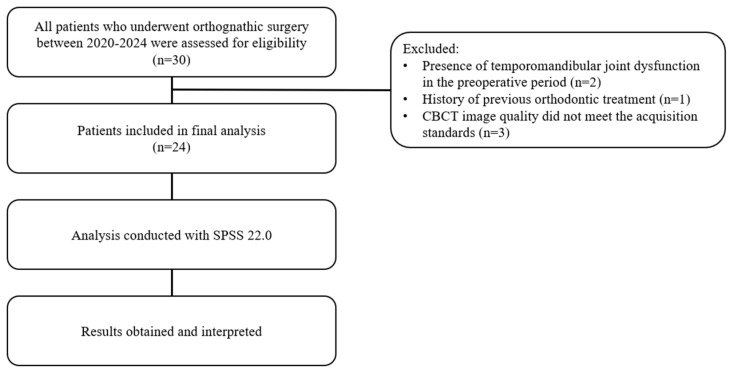
Sample selection and evaluation.

Initially, the records of 30 patients scheduled for bimaxillary orthognathic surgery were examined. However, 6 individuals were excluded because of the detection of TMJ dysfunction in the preoperative period (2 patients), a history of previous orthodontic treatment (1 patient), and inadequate CBCT image quality that did not meet scanning standards (3 patients). Consequently, the preoperative and postoperative records of 24 individuals (48 condyles) were evaluated. The patients were divided into two groups according to the presence or absence of condyle-centered mandibular CCW autorotation in the digital surgical planning data (Figure 1).

Individuals with an autorotation angle in the range of 4–7° were included in the autorotation-present group, whereas those in whom no autorotation angle was detected were assigned to the autorotation-absent group.

### 2.3. Imaging and Measurement Protocols

Cephalometric analyses were conducted using standardized lateral cephalometric radiographs and sagittal and coronal CBCT sections of the right and left temporomandibular joints obtained at the preoperative stage (T0) and at the first postoperative year (T1).

Sagittal measurements were performed on the mediolateral slice displaying the greatest width of each condyle. This plane was defined as the standard central section that includes the medial and lateral poles of the condylar head and provides the clearest visualization of condylar morphology. Slice selection was simultaneously verified using the coronal and axial planes.

**Figure 2:**
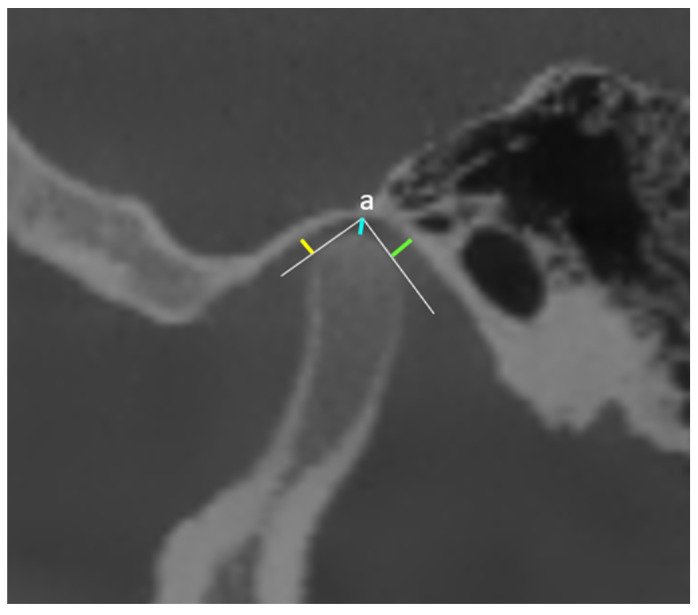
Sagittal CBCT image demonstrating the measurements of AJS (yellow line), SJS (blue line), and PJS (green line).

For the sagittal measurements, the following parameters were evaluated. The highest point of the mandibular fossa was designated point (a), which was identified by drawing two tangent lines connecting the most anterior and most posterior borders of the condylar head. Perpendicular lines were drawn to these tangents to measure the anterior joint space (AJS) and posterior joint space (PJS). Additionally, the distance between point (a) and the highest point of the condyle was defined as the superior joint space (SJS) (Figure 2).

**Figure 3:**
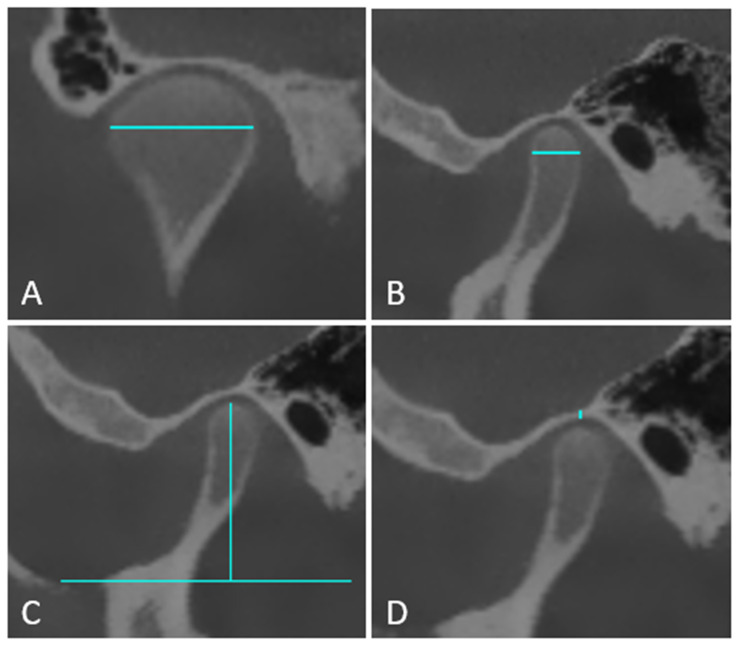
CBCT image demonstrating the measurements of condylar width (**A**), condylar depth (**B**), condylar height (**C**), and glenoid fossa thickness (**D**).

The distance between the most anterior and most posterior points of the condyle was measured as the condylar depth; the distance between the deepest point of the sigmoid notch and the highest point of the condyle was the condylar height; and the distance between the highest points of the inner and outer cortical contours of the glenoid fossa was the glenoid fossa thickness (Figure 3).

Coronal measurements were performed on the coronal plane corresponding to the midpoint of the condyle, identified via axial and sagittal planes on the slices with the greatest condylar dimensions. Four parameters were evaluated in the coronal direction:

Condylar width: Measured as the distance between the most prominent mesial and distal curvature points of the condyle (Figure 3).

Medial joint space (MJS): Measured as the shortest distance between the medial pole of the mandibular condyle and the medial wall of the glenoid fossa.

Lateral joint space (LJS): Defined as the shortest distance between the lateral pole of the mandibular condyle and the lateral wall of the glenoid fossa.

**Figure 4:**
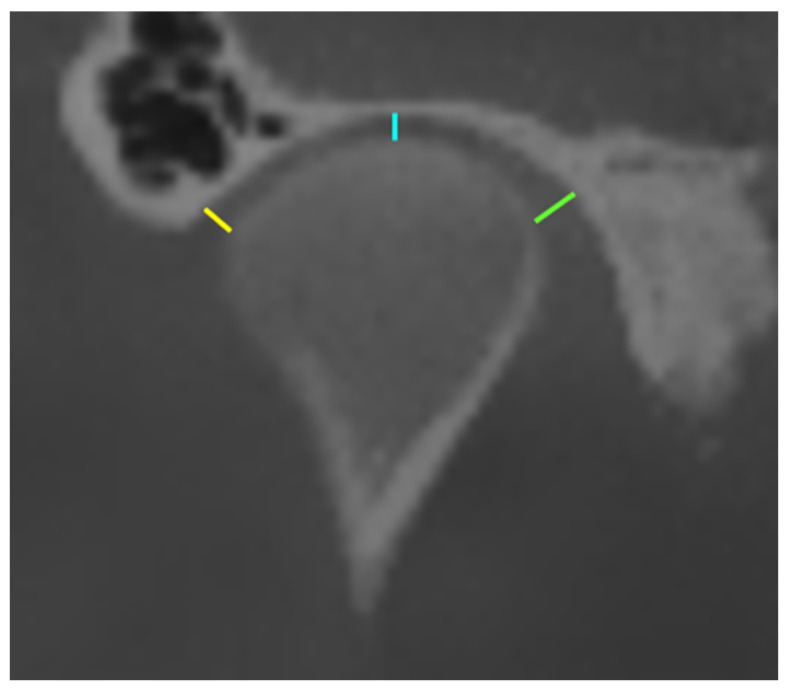
Coronal CBCT image demonstrating DJS (yellow line), CJS (blue line), and MJS (green line) measurements.

Central joint space (CJS): Defined as the shortest distance between the central surface of the mandibular condyle and the corresponding central surface of the glenoid fossa (Figure 4).

### 2.4. Surgical and Orthodontic Procedures

All surgical procedures were consistently performed by a single surgeon (M.E.Y.), whereas both preoperative and postoperative orthodontic treatments were managed by the same orthodontist (M.B.K.) for all participants. During surgery, the condylar heads were manually guided into a centered position within the glenoid fossae to achieve proper condylar seating. Previous studies have demonstrated that manual condylar positioning provides a level of accuracy comparable to computer-assisted fixation systems [16,17,18]. This standardized approach was adopted to reduce operator-dependent variability throughout both the surgical and orthodontic phases of treatment. On postoperative day two, rigid intermaxillary elastics were applied to stabilize the mandible in the intended occlusal position, and these elastics were maintained for a period of 2–3 weeks. Thereafter, lighter guiding elastics were introduced to support occlusal guidance, and patients were instructed to initiate mouth-opening exercises. The occlusal acrylic splint was removed at three weeks postoperatively. Postoperative orthodontic treatment was typically initiated between 6 and 8 weeks following surgery.

### 2.5. Measurement Reliability

All quantitative assessments were conducted by a single calibrated investigator (M.D.). To determine intra-examiner consistency, the same predefined anatomical landmarks were re-evaluated on each participant’s CBCT dataset following a two-week washout period. Reliability between the two measurement sessions was quantified using intraclass correlation coefficient analysis based on a two-way absolute agreement model. ICC values exceeding 0.90 across all parameters confirmed excellent measurement consistency and reproducibility.

### 2.6. Imaging Protocol

CBCT imaging was performed using a NewTom 7G system (Verona, Italy) with a field of view of 29 × 30 cm. Exposure settings were individually adjusted by a single experienced technician based on patient body habitus. All imaging procedures adhered to the ALARA (As Low As Reasonably Achievable) principle. Automatic exposure parameters were applied, with a slice thickness of 1 mm. Scans were acquired with patients in an upright position, and head stabilization was ensured using a dedicated support device. Standard radiation protection measures routinely used in clinical practice were implemented. Following image reconstruction with a voxel size of 0.600 mm, axial, sagittal, and coronal images were analysed using Synapse software (https://nemotec.com/, Fujifilm Corporation, Tokyo, Japan).

### 2.7. Statistical Analyses

SPSS software (version 22.0) was the main tool to perform the statistical analyses. The Shapiro–Wilk test was used to evaluate data distribution for normality. Non-parametric statistical methods were applied, as the variables did not meet the assumptions of normal distribution. Between-group comparisons were conducted using the Mann–Whitney U test, while within-group comparisons were analysed with the Wilcoxon signed-rank test. A significance level of 0.05 was adopted; *p* < 0.05 was considered indicative of a statistically significant difference, whereas *p* > 0.05 indicated no significant difference.

## 3. Results

A total of 24 individuals (48 condyles) were included in the study; 12 of them were assigned to the no-autorotation group (Group 1), and 12 were assigned to the autorotation-present group (Group 2). The mean age of the participants was similar in both groups, with values of 25.82 ± 2.39 years and 26.37 ± 5.71 years, respectively (*p* = 0.989). The proportion of females in the overall sample was 83.3%, and no significant difference was found between the groups in terms of sex distribution (*p* > 0.05) (Table 1).

Cephalometric analyses revealed no significant differences between the groups in terms of SNA, SNB, ANB, or vertical directional angle (*p* > 0.05). Menton deviation was significantly lower in the autorotation-present group (0.90 ± 1.05 mm) than in the no-autorotation group (2.96 ± 2.38 mm) (*p* = 0.019). When the surgical movements performed in each group were examined, the amount of maxillary impaction was significantly greater in the autorotation-present group (4.98 ± 1.95 mm vs. 0.44 ± 1.38 mm; *p* < 0.001). No significant differences were observed between the groups in terms of maxillary advancement, mandibular setback, or mandibular advancement (all *p* > 0.15) (Table 2).

In the preoperative (T0) measurements, no significant differences were found between the two groups regarding anterior, posterior, superior, medial, lateral, and central joint spaces; condylar depth; condylar height; condylar width; or glenoid fossa thickness (*p* > 0.05). These preoperative findings are presented in detail in Supplementary Table S1.

In the within-group comparisons, Group 1 showed significant decreases in SJS (*p* = 0.034), condylar height (*p* = 0.003), condylar depth (*p* = 0.006), glenoid fossa thickness (*p* = 0.003), and condylar width (*p* = 0.004) in the right TMJ. In the left TMJ, a marked increase was observed in the PJS (*p* = 0.021), whereas the condylar height (*p* = 0.022), glenoid fossa thickness (*p* = 0.021), and condylar width (*p* = 0.012) significantly decreased. Although most joint space measurements in the total condyle evaluation of Group 1 did not reveal significant changes, a decreasing trend was observed in the condylar dimensions, particularly in height, depth, and width.

In Group 2, the condylar height (*p* = 0.011) and width (*p* = 0.002) decreased in the right TMJ, whereas the MJS (*p* = 0.041) significantly increased. In the left TMJ, the condylar height (*p* = 0.021), condylar depth (*p* = 0.049), and condylar width (*p* = 0.002) significantly decreased. No significant differences were observed in the remaining parameters (*p* > 0.05). In the total condyle evaluation of Group 2, changes in joint spaces were limited, whereas notable reductions in condylar morphology were observed.

In the between-group comparisons, no significant differences were found in joint space or glenoid fossa thickness (*p* > 0.05), except for the change in right SJS, which was significantly different (*p* = 0.024). In the no-autorotation group, this space decreased by an average of −0.34 ± 0.51 mm, whereas in the autorotation group, it increased by +0.19 ± 0.69 mm. No statistically significant differences were detected in the other Δ-parameters (*p* > 0.05). Detailed intergroup comparisons of postoperative changes (Δ-parameters) are provided in Supplementary Table S2.

## 4. Discussion

One of the most common concerns regarding the preservation of stability and maintenance of patient comfort after bimaxillary orthognathic surgery is TMJ dysfunction. The jaw movements planned during the surgical planning process and the intraoperative repositioning of the bone segments may affect the TMJ, potentially leading to changes in the mandibular condyle position [19].

The correction of vertical problems or the management of mandibular deficiency typically involves superior repositioning of the maxilla through Le Fort I osteotomy, which consequently results in mandibular autorotation [20]. When the surgical movements performed in our study were evaluated, maxillary impaction was found to be statistically significant in the autorotation group.

There are numerous studies in the literature regarding condylar morphological changes following orthognathic surgery. In their study of Class III patients, Gülcek et al. reported that, after bimaxillary orthognathic surgery, reductions occurred in the condylar dimensions in all three planes—width, height, and depth [21]. In studies conducted on patients with skeletal Class III malocclusion, a marked decrease in condylar height has been reported in both sagittal and coronal sections following surgery [12,22]. Additionally, medial rotation of the condylar axial angle and new bone formation in the anteromedial region have been identified. Ueki et al. examined postoperative changes across different skeletal malocclusion classes together with variations in bite forces and reported that bite force did not return to normal occlusal levels in either group and that reductions in condylar dimensions were observed [23]. In a review focusing on TMJ dysfunction, it was reported that orthognathic surgery has few to no adverse effects on TMJ morphology [24]. In our study, decreases in fossa thickness as well as reductions in condylar height, width, and depth were observed in both groups following orthognathic surgery. These findings, which are consistent with the literature, suggest that the TMJ movements occurring between the surgical groups—and the implementation of autorotation—do not affect the condylar morphology that exceeds the adaptive capacity of the joint.

Although numerous studies have examined changes in joint spaces following orthognathic surgery, the question of whether this surgical intervention alters the relationships among the structures within the TMJ complex has not yet been definitively answered [25]. Ravelo et al. reported that sagittal plane measurements taken six months after orthognathic surgery revealed significant differences in joint spaces depending on the skeletal malocclusion class. In their study, AJS increased, whereas SJS and PJS decreased in Class II patients; on the other hand, in Class III patients, AJS and SJS decreased, and PJS increased. These findings indicate that following surgery, the condyle tends to move upwards and backwards in Class II patients and upwards and forwards in Class III patients [26]. These findings are also consistent with the results reported by Abbasi et al. and da Silva et al. [27,28]. Vogl et al., in their study on Class II and Class III patients with DFDs, reported statistically significant decreases in the MJS, LJS, and SJS in the postoperative period. These authors reported that these changes in joint spaces were associated with superior movement of the condyle within the mandibular fossa and that this upward movement led to reductions in both the medial and lateral joint spaces [1]. In our study, a decrease in SJS and glenoid fossa thickness and an increase in PJS were observed in the group without autorotation. This finding, which is consistent with the literature, indicates that the condyle moves in a superior direction. This movement may trigger a remodeling process in the glenoid fossa, leading to a reduction in fossa thickness. This superior displacement is considered a reflection of the physiological adaptation of the proximal segment to the new occlusal scheme following sagittal-plane surgical movements and is associated with the condyle repositioning itself within the fossa into a more superior and commonly accepted stable position. This situation is also consistent with the findings of a CBCT-based study reporting a decrease in condylar height and resorptive remodeling in the anterosuperior region following BSSRO [22].

Zhang et al., in their study of asymmetric cases, reported significant differences between the nondeviated and deviated sides in terms of the sagittal ramus angle and medial and lateral joint spaces during the preoperative period. This finding suggests that facial asymmetry may lead to angular and joint space asymmetry within the TMJ. Following BSSRO, however, no significant differences remained between the two TMJ sides for any of the morphological variables, and the researchers stated that BSSRO can correct TMJ morphological asymmetry in patients with facial asymmetry [29]. Similarly, Roman et al., in their study of patients with skeletal Class III malocclusion, reported isolated significant findings and observed an increase in MJS [30]. In our study, while no significant difference was observed in the left-side MJS within the autorotation group, an increase was noted in the right-side MJS. This finding may be attributed to the correction of mandibular asymmetry.

A review of the existing literature indicates that reported dimensional changes in the SJS span a wide range, from −0.62 mm to +2.66 mm [1,26,29,31,32,33,34,35,36]. In the present study, intergroup comparisons demonstrated a statistically significant difference in SJS, attributable to a reduction in SJS in the non-autorotation group, whereas no significant change was observed in the autorotation group. Nevertheless, the magnitude, direction, and clinical relevance of joint space alterations appear to be influenced by multiple factors, including the specific surgical approach employed and the patients’ individual adaptive capacity, and their clinical implications remain incompletely defined in the literature [25]. In this context, the present findings suggest that incorporating condyle-centered autorotation into surgical planning may help preserve SJS morphology during the early postoperative adaptation period.

It should be acknowledged that the autorotation group showed significantly greater maxillary impaction and lower preoperative menton deviation, both of which may act as potential confounding factors. Superior repositioning of the maxilla is known to induce mandibular autorotation and may influence condylar loading and joint space adaptation [6,7]. However, other planned surgical movements were comparable between groups. Despite greater maxillary impaction, the autorotation group did not exhibit SJS reduction, whereas a decrease was observed in the non-autorotation group, suggesting that factors beyond maxillary impaction alone may influence postoperative condyle-fossa adaptation. Nevertheless, due to the retrospective design and limited sample size, the independent effects of maxillary movement and autorotation cannot be fully separated and should be considered a limitation.

In BSSRO-based mandibular surgery, the impact of pitch rotation of the proximal ramus segment on relapse remains controversial, as both positive [37,38] and negative [39,40] correlations have been reported in the literature. While surgical simulation guidelines recommend keeping the pitch rotation of this segment within 4° and the roll rotation within 3° [41], Wu et al. reported that even in asymmetric Class III cases with clockwise or counterclockwise rotations reaching approximately 7°, stable occlusion and preserved TMJ function were maintained during the 1-year follow-up period [42]. Pachnicz and Strózyk, however, reported that changes in masticatory muscle force were minimal when the rotation angle remained below 3° [43]. In this context, the 4–7° range of CCW rotation examined in our study provides unique insights into changes in condylar morphology and joint spaces compared with studies in the literature that have investigated smaller rotational values.

In studies focusing on postoperative TMJ adaptation, Kim et al. evaluated condylar position at 6- and 12-month follow-ups after bimaxillary surgery and reported that it became relatively stable after six months [44]. Tabrizi et al. assessed changes in condylar position in patients with a vertical maxillary growth pattern and reported that the condyles showed an adaptation approximately to their initial positions nine months after surgery [35]. In addition, Chen et al. demonstrated that the condyle began to return to a concentric position by the third postoperative month and remained stable at the end of the first year [34]. Firoozei et al., in their study involving 15 patients, performed magnetic resonance imaging before orthognathic surgery and at the third postoperative month. They reported that the preoperative disc position was 5.74 ± 1.21 and the postoperative position was 5.65 ± 1.06, concluding that no significant short-term change in the condyle–disc ratio was observed [45]. Eshghpour et al. reported an average anterior disc displacement of 0.32 mm following orthognathic surgery in individuals with mandibular retrognathia who underwent less than 5 mm of mandibular advancement. They noted that this finding may be associated with changes occurring in the condylar position and its relationship with the fossa [46]. In our study, changes occurring in the TMJs that maintained a stable position preoperatively and during the first postoperative year were evaluated. No statistically significant difference was found between the groups in terms of the number of surgical movements performed during orthognathic surgery. Similarly, when the preoperative and postoperative periods were compared, no significant differences were observed in the joint space. These findings indicate that in patients who underwent 4–7° CCW rotation, the TMJ was able to maintain a stable position during the first postoperative year. However, the discal tissues of the TMJ were not examined during this process, which is one of the limitations of our study.

The relationship between dental occlusion and temporomandibular disorders has long been a subject of debate. However, the current literature does not support a direct or unidirectional causal relationship between occlusion and temporomandibular disorders [47]. As a natural consequence of orthognathic surgery, both occlusion and mandibular position are altered, and the subsequent adaptive responses observed in the TMJ represent a multifactorial process that extends beyond condylar morphology to include neuromuscular, biological, and psychosocial components [48,49]. Structural findings identified through imaging modalities, such as changes in joint space or condylar remodeling, do not inherently indicate the presence of functional impairment or pathology [25]. In this context, and in line with current consensus [50], the radiographic findings of the present study should be interpreted not as evidence of a deterministic association between occlusal or mandibular positional changes and TMJ pathology, but rather as reflections of postoperative structural adaptation.

This study is one of the few investigations that have quantitatively and three-dimensionally evaluated the effect of mandibular autorotation on TMJ morphology via CBCT data. The measurements were obtained via high-resolution standardized protocols and validated via repeatability analysis performed by a single researcher (ICC > 0.90). The surgical procedures were carried out by the same surgeon, and the orthodontic treatments were performed by the same orthodontist, thereby minimizing operator-related variation. In addition, grouping the participants according to the autorotation angle enabled the clinically meaningful analysis of a parameter that directly influences the condyle–fossa relationship.

Although the sample size of our study is somewhat limited, evaluating this specific patient group is clinically valuable for understanding condylar adaptation. However, the analysis was restricted to skeletal parameters only; the position of the articular disc, intra-articular soft tissues, muscle forces, and dynamic functional joint behavior could not be evaluated due to the absence of magnetic resonance imaging and functional assessments. In addition, functional outcomes such as pain, joint sounds, range of motion, and patient-reported symptoms were not included, precluding conclusions regarding clinical TMJ function. Furthermore, the retrospective design limits the ability to establish causal relationships between mandibular autorotation, occlusal changes, and temporomandibular joint health and makes it difficult to fully control for individual biomechanical adaptation following surgery. Finally, significant preoperative differences between the groups, including menton deviation and the magnitude of maxillary impaction, should be acknowledged as potential confounding factors that may have influenced postoperative TMJ adaptation and the interpretation of the results. Therefore, future prospective studies with larger sample sizes, incorporating multimodal imaging and standardized functional outcome measures, are needed to enhance the generalizability and interpretative scope of the present findings.

## 5. Conclusions

This study demonstrated that condyle-centered CCW mandibular autorotation performed during bimaxillary orthognathic surgery leads to limited yet regionally significant changes in TMJ morphology. In both groups, reductions in condylar height, condylar width, and glenoid fossa thickness were observed, indicating an adaptive bone remodeling process. However, in the autorotation group, the superior joint space was preserved or slightly increased, whereas a decrease was observed in the group without autorotation. This finding suggests that autorotation may help maintain the condyle–fossa relationship within more physiological limits, thereby reducing compressive loading on the articular structures. Consequently, incorporating mandibular autorotation into surgical planning may be an important factor in improving biomechanical balance, joint stability, and long-term functional outcomes.

## Figures and Tables

**Table 1:** Age and sex of the study participants.

		Group 1 n = 12	Group 2 n = 12	Total n = 24
Age (Year)	Mean ± SD	25.82 ± 2.39	26.37 ± 5.71	26.09 ± 4.29
Sex	Male	3 (25.0%)	1 (8.3%)	4 (16.7%)
	Female	9 (75.0%)	11 (91.7%)	20 (83.3%)

n: number of participants.

**Table 2:** Preoperative cephalometric analysis data and planned jaw movements.

Parameter	Group 1 (Mean ± SD)	Group 2 (Mean ± SD)	*p*
SNA (°)	81.17 ± 2.66	80.92 ± 4.52	0.989
SNB (°)	81.25 ± 5.12	77.08 ± 6.71	0.283
ANB (°)	−0.08 ± 3.80	3.83 ± 6.21	0.111
Vertical direction (°)	35.50 ± 7.29	38.67 ± 5.37	0.213
Menton deviation (mm)	2.96 ± 2.38	0.90 ± 1.05	0.019
Maxillary impaction (mm)	0.44 ± 1.38	4.98 ± 1.95	0.0001 *
Maxillary advancement (mm)	4.00 ± 1.97	3.58 ± 1.63	0.630
Right mandibular setback (mm)	1.98 ± 1.85	1.94 ± 2.32	0.719
Left mandibular setback (mm)	2.32 ± 2.09	1.77 ± 2.22	0.549
Right mandibular advancement (mm)	1.24 ± 2.12	3.50 ± 3.93	0.156
Left mandibular advancement (mm)	1.47 ± 2.66	2.87 ± 3.69	0.367

*p*: Mann—Whitney U test; *: Statistically significant.

## Data Availability

The data presented in this study are available on request from the corresponding author due to ethical restrictions.

## Supplementary Materials

The following supporting information can be downloaded at: https://www.mdpi.com/article/10.3390/jcm15031296/s1, Table S1. Comparison of Condylar and Fossa Parameters Measured in the TMJ Between Groups in the Preoperative (T0) Period; Table S2. Comparison of preoperative (T0) and postoperative (T1) condylar and fossa measurement values by group.

## Abbreviations

The following abbreviations are used in this manuscript:

AJS	Anterior Joint Space
ALARA	As Low As Reasonably Achievable
ANB	A point-Nasion-B point angle
BSSRO	Bilateral Sagittal Split Ramus Osteotomy
CBCT	Cone-Beam Computed Tomography
CCW	Counterclockwise
CJS	Central Joint Space
CT	Computed Tomography
DFD	Dentofacial Deformity
DJS	Distal Joint Space
FOV	Field of View
ICC	Intraclass Correlation Coefficient
LJS	Lateral Joint Space
MRI	Magnetic Resonance Imaging
MJS	Medial Joint Space
PJS	Posterior Joint Space
SJS	Superior Joint Space
SNA	Sella-Nasion-A point angle
SNB	Sella-Nasion-B point angle
TMJ	Temporomandibular Joint
