# Supplementary material for: Effect of Counterclockwise Mandibular Autorotation on Temporomandibular Joint Spaces and Condylar Morphology After Bimaxillary Orthognathic Surgery: A CBCT-Based Study
Source: J Clin Med. 2026 Feb 6;15(3):1296. doi: 10.3390/jcm15031296 (PMC12898013; doi:10.3390/jcm15031296)
Supplement: Supplementary file 1 [file jcm-15-01296-s001.zip › Supplementary Table S1.pdf]

Supplementary Table S1. Comparison of Condylar and Fossa Parameters Measured in the TMJ Between Groups in the Preoperative (T0) Period

T0		Group 1 (mean ± SD)	Group 2 (mean ± SD)	p
AJS	Right	2.10 ± 0.71	2.20 ± 0.28	0.101
	Left	2.20 ± 0.56	2.08 ± 0.28	0.931
	Total	2.15 ± 0.63	2.14 ± 0.28	0.297
PJS	Right	1.98 ± 0.35	2.05 ± 0.60	0.563
	Left	2.13 ± 0.70	2.03 ± 0.60	0.931
	Total	2.06 ± 0.54	2.04 ± 0.59	0.805
SJS	Right	2.12 ± 0.48	2.19 ± 0.44	0.402
	Left	2.09 ± 0.57	2.16 ± 0.24	0.436
	Total	2.10 ± 0.51	2.18 ± 0.35	0.283
MJS	Right	2.16 ± 0.75	2.11 ± 0.63	0.977
	Left	2.23 ± 0.71	2.11 ± 0.45	0.908
	Total	2.20 ± 0.72	2.11 ± 0.54	0.853
CJS	Right	1.85 ± 0.54	2.10 ± 0.60	0.341
	Left	2.44 ± 1.16	2.03 ± 0.28	0.623
	Total	2.15 ± 0.94	2.06 ± 0.46	0.628
DJS	Right	1.91 ± 0.86	1.89 ± 0.68	0.817
	Left	2.04 ± 0.64	1.87 ± 0.81	0.525
	Total	1.97 ± 0.75	1.88 ± 0.73	0.665
Condylar Height	Right	17.02 ± 2.61	16.39 ± 2.18	0.525
	Left	16.62 ± 2.90	16.45 ± 2.29	0.977
	Total	16.82 ± 2.70	16.42 ± 2.19	0.297
Condylar Depth	Right	7.12 ± 1.28	7.29 ± 0.97	0.261
	Left	7.09 ± 1.34	7.41 ± 1.30	0.157
	Total	7.10 ± 1.28	7.35 ± 1.12	0.103
Condylar Width	Right	15.75 ± 2.07	16.58 ± 2.90	0.471
	Left	15.34 ± 1.84	15.97 ± 2.28	0.795
	Total	15.54 ± 1.93	16.27 ± 2.57	0.458
Glenoid Fossa Thickness	Right	1.69 ± 0.23	1.76 ± 0.31	0.386
	Left	1.66 ± 0.18	1.74 ± 0.53	0.564
	Total	1.67 ± 0.20	1.75 ± 0.42	0.288

p: Mann–Whitney U test.
