# Supplementary material for: Effect of Counterclockwise Mandibular Autorotation on Temporomandibular Joint Spaces and Condylar Morphology After Bimaxillary Orthognathic Surgery: A CBCT-Based Study
Source: J Clin Med. 2026 Feb 6;15(3):1296. doi: 10.3390/jcm15031296 (PMC12898013; doi:10.3390/jcm15031296)
Supplement: Supplementary file 1 [file jcm-15-01296-s001.zip › Supplementary Table S2.pdf]

Supplementary Table S2. Comparison of preoperative (T0) and postoperative (T1) condylar and Fossa measurement values by group

		Group 1 (n=12)			Group 2 (n=12)			Group 1	Group 2	
		T0 (mean ±SD)	T1 (mean ±SD)	p*	T0 (mean±SD)	T1 (mean ±SD)	p*	T1-T0 (mean ±SD)	T1-T0 (mean ±SD)	p**
AJS	Right	2.10 ± 0.71	1.83±0.57	0.182	2.20 ± 0.28	2.10 ± 0.53	0.638	-0.27 ± 0.51	-0.10 ± 0.55	0.471
	Left	2.20 ± 0.56	2.32 ± 0.59	0.481	2.08 ± 0.28	1.82 ± 0.67	0.131	0.12 ± 0.62	-0.26 ± 0.53	0.126
	Total	2.15 ± 0.63	2.07 ± 0.62	0.617	2.14 ± 0.28	1.96 ± 0.61	0.121	-0.07 ± 0.59	-0.18 ± 0.53	0.509
PJS	Right	1.98 ± 0.35	1.87 ± 0.55	0.108	2.05 ± 0.60	2.08 ± 0.69	0.531	-0.11 ± 0.44	0.03 ± 0.81	0.214
	Left	2.13 ± 0.70	2.52 ± 0.97	0.021	2.03 ± 0.60	1.86 ± 0.65	0.844	0.39 ± 0.55	−0.17 ± 0.80	0.119
	Total	2.06 ± 0.54	2.20 ± 0.84	0.291	2.04 ± 0.59	1.97 ± 0.67	0.753	0.14 ± 0.55	-0.07 ± 0.79	0.853
SJS	Right	2.12 ± 0.48	1.78 ± 0.54	0.034	2.19 ± 0.44	2.38 ± 0.65	0.239	−0.34 ± 0.51	0.19 ± 0.69	0.024
	Left	2.09 ± 0.57	2.32 ± 0.81	0.195	2.16 ± 0.24	2.00 ± 0.59	0.424	0.24 ± 0.66	−0.17 ± 0.59	0.133
	Total	2.10 ± 0.51	2.05 ± 0.73	0.587	2.18 ± 0.35	2.19 ± 0.64	0.831	-0.05 ± 0.64	0.01 ± 0.65	0.564
MJS	Right	2.16 ± 0.75	2.18 ± 0.63	0.814	2.11 ± 0.63	2.44 ± 0.90	0.041	0.01 ± 0.56	0.33 ± 0.47	0.119
	Left	2.23 ± 0.71	2.45 ± 1.01	0.433	2.11 ± 0.45	2.33 ± 0.71	0.158	0.22 ± 0.78	0.22 ± 0.55	0.624
	Total	2.20 ± 0.72	2.31 ± 0.83	0.689	2.11 ± 0.54	2.38 ± 0.79	0.015	0.12 ± 0.67	0.27 ± 0.51	0.152
CJS	Right	1.85 ± 0.54	2.05 ± 0.74	0.456	2.10 ± 0.60	2.27 ± 0.76	0.875	0.20 ± 0.86	0.18 ± 0.62	0.908
	Left	2.44 ± 1.16	2.41 ± 0.82	0.814	2.03 ± 0.28	2.44 ± 0.63	0.051	−0.03 ± 0.86	0.41 ± 0.55	0.214
	Total	2.15 ± 0.94	2.23 ± 0.78	0.466	2.06 ± 0.46	2.36 ± 0.69	0.097	0.08 ± 0.85	0.29 ± 0.59	0.458
DJS	Right	1.91 ± 0.86	1.85 ± 0.84	0.875	1.89 ± 0.68	2.05 ± 0.71	0.388	−0.06 ± 0.36	0.16 ± 0.69	0.326
	Left	2.04 ± 0.64	2.22 ± 0.71	0.423	1.87 ± 0.81	1.83 ± 0.93	0.594	0.18 ± 0.87	−0.04 ± 0.83	0.341
	Total	1.97 ± 0.75	2.03 ± 0.79	0.758	1.88 ± 0.73	1.94 ± 0.82	0.843	0.06 ± 0.66	0.06 ± 0.75	0.893
Condylar Height	Right	17.02 ± 2.61	16.77 ± 2.62	0.003	16.39±2.18	1.85 ± 2.44	0.011	−0.26 ± 0.29	−0.54 ± 0.64	0.419
	Left	16.62 ± 2.90	15.87 ± 2.32	0.022	16.45±2.29	16.22 ± 2.34	0.021	−0.75 ± 1.59	−0.23 ± 0.30	0.773
	Total	16.82 ± 2.70	16.32 ± 2.46	0.0001	16.42±2.19	16.03 ± 2.35	0.001	-0.51 ± 1.14	-0.39 ± 0.51	0.934
Condylar Depth	Right	7.12 ± 1.28	6.89 ± 1.40	0.060	7.29 ± 0.97	7.08 ± 1.40	0.182	−0.23 ± 0.32	−0.22 ± 0.54	0.707
	Left	7.09 ± 1.34	6.98 ± 1.76	0.531	7.41 ± 1.30	7.18 ± 1.39	0.049	−0.10 ± 1.25	−0.23 ± 0.54	0.707
	Total	7.10 ± 1.28	6.94 ± 1.56	0.025	7.35 ± 1.12	7.13 ± 1.36	0.023	-0.17 ± 0.89	-0.23 ± 0.53	0.842
Condylar Width	Right	15.75 ± 2.07	15.38 ± 2.18	0.004	16.58±2.90	16.15 ± 2.61	0.002	−0.36 ± 0.32	−0.43 ± 0.49	0.977
	Left	15.34 ± 1.84	14.09 ± 3.24	0.012	15.97±2.28	14.59 ± 2.71	0.002	−1.25 ± 1.87	−1.38 ± 1.95	0.564
	Total	15.54 ± 1.93	14.73 ± 2.78	0.0001	16.27±2.57	15.37 ± 2.72	0.0001	-0.81 ± 1.39	-0.90 ± 1.47	0.529
Glenoid Fossa Thickness	Right	1.69 ± 0.23	1.62 ± 0.24	0.003	1.76 ± 0.31	1.83 ± 0.53	0.937	−0.07 ± 0.04	0.07 ± 0.33	0.132
	Left	1.66 ± 0.18	1.52 ± 0.21	0.021	1.74 ± 0.53	1.69 ± 0.40	0.288	−0.14 ± 0.22	−0.05 ± 0.35	0.401
	Total	1.67 ± 0.20	1.57 ± 0.23	0.0001	1.75 ± 0.42	1.76 ± 0.46	0.431	-0.10 ± 0.16	0.01 ± 0.34	0.101

* Wilcoxon signed-rank test ** Mann–Whitney U test.
